# Phloridzin, an Apple Polyphenol, Exerted Unfavorable Effects on Bone and Muscle in an Experimental Model of Type 2 Diabetes in Rats

**DOI:** 10.3390/nu10111701

**Published:** 2018-11-07

**Authors:** Piotr Londzin, Szymon Siudak, Urszula Cegieła, Maria Pytlik, Aleksandra Janas, Arkadiusz Waligóra, Joanna Folwarczna

**Affiliations:** Department of Pharmacology, School of Pharmacy with the Division of Laboratory Medicine in Sosnowiec, Medical University of Silesia, 40-055 Katowice, Poland; piotr.londzin@vp.pl (P.L.); szymons1992@interia.pl (S.S.); ucegiela@o2.pl (U.C.); mariapytlik@gmail.com (M.P.); ajanas@sum.edu.pl (A.J.); arekwaligora@gmail.com (A.W.)

**Keywords:** phloridzin, diabetes, osteoporosis, rats, skeletal system, skeletal muscle

## Abstract

It is believed that apple fruits contain components with health-promoting effects, including some antidiabetic activity. One of the most known apple compounds is phloridzin, a glucoside of phloretin. Phloridzin and phloretin were reported to exert some favorable skeletal effects in estrogen-deficient rats and mice. The aim of the study was to investigate the effects of phloridzin on musculoskeletal system in rats with type 2 diabetes induced by a high-fat diet (HFD) and streptozotocin (STZ). The experiments were performed on mature female Wistar rats, divided into control rats (fed a standard laboratory diet), HFD/STZ control rats, and HFD/STZ rats receiving phloridzin (20 or 50 mg/kg/day per os) for four weeks. Serum biochemical parameters, muscle mass and strength, bone mass, density, histomorphometric parameters and mechanical properties were determined. The HFD/STZ rats developed hyperglycemia, with decreases in the muscle mass and strength and profound osteoporotic changes. Phloridzin at 20 mg/kg markedly augmented the unfavorable effects of diabetes on the muscle mass and strength and decreased growth of bones, whereas, at 50 mg/kg, it did not affect most of the investigated musculoskeletal parameters. Results of the study indicate the possibility of unfavorable effects of phloridzin on the musculoskeletal system in conditions of hyperglycemia.

## 1. Introduction

It is commonly believed that apple (Malus × domestica Borkh. fruit) consumption may be associated with a decreased risk of cancer, cardiovascular diseases or type 2 diabetes [1,2,3,4,5], because apples are a rich source of many compounds, such as flavonoids, phenolic acids, vitamins, that have health-promoting effects. One of the most recognized apple polyphenols is phloridzin (phlorizin). Phloridzin is a glycoside of phloretin (phloretin 2′-O-glucoside), a flavonoid belonging to the chemical class of dihydrochalcones [6,7]. Phloridzin is mainly present in apple leaves and bark, but also, in lower amounts, fruits [3,6,7]. Unpeeled apples are a richer source of phloridzin than peeled ones, since phloridzin content in apple peel is 12–418 mg/kg, whereas in apple pulp—4–20 mg/kg [6]. Moreover, old apple cultivars contain bigger amounts of phloridzin than newer ones [8].

Phloridzin was first isolated from the apple tree bark by De Koninck in 1835 [7]. The most intensively studied were the effects of phloridzin on glucose absorption and excretion, and diabetes [6,7,9]. Phloridzin has become the precursor of a new class of antidiabetic drugs—SGLT2 (sodium-glucose cotransporter 2) inhibitors [6,10].

SGLT cotransporters are membrane proteins involved in the sodium-dependent glucose transport, especially the absorption of glucose in the small intestine (SGLT1) and the reabsorption of glucose from the glomerular filtrate in the kidneys (SGLT2 and, to a lesser extent, SGLT1) [11,12]. Phloridzin inhibits competitively SGLT1 and SGLT2 [6,13]. Blockade of SGLT1 leads mainly to inhibition of glucose absorption from the small intestine as a result of the reduction of active transcellular glucose uptake [14]. Blockade of SGLT2 inhibits glucose reabsorption in the kidneys, increasing its excretion [11,12]. Both the effects on glucose absorption and glucose excretion decrease serum blood glucose concentration. Phloridzin, however, is a glycoside that is broken down by lactase hydrolase in the brush border membrane of the small intestine epithelial cells to phloretin and glucose [6,14]. Therefore, after oral administration, phloridzin may inhibit only SGLT1 leading to a decrease of gut glucose absorption; its other activities result from the activity of the aglycone—phloretin. Phloretin is a much weaker inhibitor of SGLT1 than phloridzin [14], but it blocks GLUT2, a transporter responsible for removing glucose from enterocytes into the circulation [14,15], and GLUT1, responsible for glucose uptake in various tissues (which may affect glucose utilization) [16]. However, also after oral administration, phloridzin has been reported to exert beneficial effects in experimental diabetic animals [9,17,18,19]. In addition, the hypoglycemic activity of powdered unripe apples (in a dose of about 315 mg of phloridzin) or apple extract (about 450 mg of phloridzin) has been demonstrated in humans [14,20].

Diabetes is a chronic, progressive disease that affects more than 420 million people in the world [21]. Numerous complications of diabetes include nephropathy, neuropathy, retinopathy, and increased risk of fractures, to which sarcopenia may contribute [22,23]. There are two main types of diabetes; the most common, including about 90–95% of diabetic patients, is type 2 diabetes (T2D) [24]. Both T1D and T2D induce disorders in bone and muscle metabolism that may result, among others, from insulin deficiency as well as insulin resistance [25,26]. Patients with T1D usually show a decrease in bone mineral density (BMD) and muscle mass, whereas, in patients with T2D, such changes are not observed or there are even increases in BMD and muscle mass [25,26]. Nevertheless, in both types of diabetes, the fracture risk is increased [26]. Moreover, there is increasing body of evidence that antidiabetic drugs themselves may affect the skeletal system and fracture rate [23]. Also, dietary factors may affect the development of musculoskeletal disorders in diabetes.

There are very few data about the impact of phloridzin on the skeletal system in experimental conditions, and there are no data concerning its impact on bones in humans. The skeletal effects of phloridzin in experimental models of diabetes have never been reported. However, it was demonstrated that high intake of phloridzin (0.25% in the diet for 80 days) favorably affected the skeletal system of rats with estrogen deficiency and inflammation, counteracting the development of skeletal changes induced by the procedures [27]. Very recently, phloridzin and phloretin (both at 10 mg/kg per os (p.o.) for 12 weeks) have been reported to counteract the development of senile osteoporosis in mice through increasing bone formation and mineralization, and inhibiting bone loss [28]. Phloretin, which may exert weak estrogenic activity [29,30], inhibited osteoclast formation, activation and survival in vitro [31,32,33], affected function of osteocytic MLO-Y4-A2 cells in vitro [34], and, at 10 mg/kg p.o. for eight weeks, counteracted the development of osteoporosis in estrogen-deficient mice in vivo [31].

We were interested in effects of phloridzin on the development of changes in the musculoskeletal system of rats with experimental type 2 diabetes. The available rat models of type 2 diabetes do not fully reproduce human type 2 diabetes [35]. One of type 2 diabetes models is a model of diabetes induced by a high-fat diet (HFD) and streptozotocin (STZ), administered two weeks after introduction of HFD [36,37]. The combination of the diet and a single administration of streptozotocin, which selectively accumulates in pancreatic β-cells and destroys them, was designed to reproduce the pathology of this type of diabetes in humans [36]. Phloridzin was used at doses of similar range to reported by others to affect glycemia in experimental animals [17,18].

The research hypothesis of the present study was that phloridzin may affect the musculoskeletal system of rats with type 2 diabetes. The aim of the study was to investigate the effects of phloridzin at doses of 20 and 50 mg/kg p.o. daily for four weeks on the musculoskeletal system of Wistar rats with experimental HFD/STZ diabetes [36,37]. The bone and muscle mass and strength, bone density, composition and microstructure, as well as the serum bone turnover markers, were investigated in the diabetic rats, and the results were compared with those of the diabetic and healthy controls. Moreover, in order to confirm the development of diabetes and to investigate the effects of phloridzin on diabetic rats, selected serum biochemical parameters concerning mainly carbohydrate and lipid metabolism were measured.

## 2. Materials and Methods

### 2.1. Animals and Chemicals

The experiments were carried out on mature female Wistar rats (*Rattus norvegicus*) obtained from the Center of Experimental Medicine, Medical University of Silesia, Katowice, Poland, at the age of 13–14 weeks. The animals were maintained under monitored standard laboratory conditions complying to the European Union guidelines (directive 2010/63/EU), in standard plastic cages (Tecniplast, Buguggiate, Italy), 5–6 rats per cage, under a 12-h light–12-h dark cycle (light on 7:00 a.m.). The experiments were approved by the Local Ethics Commission, Katowice, Poland (permission numbers: 37/2015 and 3/2017). The experiment started after one-week acclimatization.

The drugs used in the experiments included: phloridzin dihydrate at doses of 21.6 and 54.1 mg/kg p.o., i.e., 20 and 50 mg/kg p.o. of phloridzin (Sigma-Aldrich Co., St. Louis, MO, USA); streptozotocin (Cayman Chemical Company, Ann Arbor, MI, USA); a drug used in order to mark the calcification front: tetracycline hydrochloride (Sigma-Aldrich Co., St. Louis, MO, USA); drugs used for general anesthesia: ketamine-Ketamina 10% (Biowet Puławy Sp. z o. o., Puławy, Poland) and xylazine-Sedazin (Biowet Puławy Sp. z o. o., Puławy, Poland).

The body mass of rats ranged between 188–237 g on the start of the experiment. The rats were originally allocated to four experimental groups (*n* = 10–12 per group) based on their body mass, so that the mean body mass in all groups was equal at the start of the experiment. The number of rats per group was chosen based on previous experiments concerning the skeletal system [27,31,38,39,40]. There were following experimental groups: control rats (group I), and three groups with HFD/STZ diabetes: HFD/STZ control rats (group II), and HFD/STZ rats receiving orally phloridzin at doses of 20 mg/kg (group III) or 50 mg/kg (group IV).

All animals were weighed at the start of the experiment and then once a week, and additionally on the day before the end of experiment. Blood glucose level was measured in all groups, once a week, using an Accu-Chek Performa Nano glucometer (Roche Diagnostics, Mannheim, Germany) and Accu-Chek Performa test strips (Roche Diabetes Care, Mannheim, Germany). The blood samples for the measurement were taken from tail vessels of conscious rats (by cutting the tail tip).

Control rats (group I) were fed a standard laboratory diet (Labofeed B, Wytwórnia Pasz “Morawski”, Kcynia, Poland). The rats of groups II-IV were switched from the standard laboratory diet (Labofeed B) to the HFD (Labofeed B 32% fat, Wytwórnia Pasz “Morawski”, Kcynia, Poland) at the start of the experiment (2 weeks before the STZ administration) and maintained on the HFD to the end of the experiment. Two weeks after the introduction of HFD, a single dose of streptozotocin (40 mg/kg, intraperitoneally (i.p.)), dissolved in 0.1 M citrate buffer, was administered to rats of groups II-IV. Control rats (group I) received the citrate buffer in the same volume of 1 mL/kg i.p. One week after the streptozotocin administration, the development of diabetes was confirmed, based on the measurement of the non-fasting blood glucose concentration; rats with glucose levels above 300 mg/100 mL were considered diabetic. In order to obtain more similar mean blood glucose levels in the HFD/STZ groups at the start of phloridzin administration, four rats were relocated between groups. Administration of phloridzin (once daily by oral gavage, in the morning hours) started 1 week after the STZ injection and lasted 4 weeks. Phloridzin was administered as a tap water suspension (prepared with the addition of Tween 20, quantum satis) at a volume of 2 mL/kg. Control rats received the vehicle at the same volume. The four-week period of phloridzin administration was long enough to observe effects of other compounds of plant origin on the skeletal system in rats [38,39,40,41].

To mark the calcification front, tetracycline hydrochloride was administered twice at a dose of 20 mg/kg i.p. (at the start and at the end of the phloridzin administration).

One rat (from group III) did not develop diabetes and was excluded from the experiment. Seven diabetic rats died during the experiment: four rats from group II, one rat from group III and two rats from group IV. All deaths occurred 6–19 days after the start of the vehicle or phloridzin administration. The autopsy demonstrated that the colon was filled with very hard fecal masses, which probably resulted in complete obstruction of the gastrointestinal tract and led to death. The final number of rats in experimental groups at the end of the study was as follows: *n* = 11 (group I), *n* = 8 (group II), *n* = 9 (group III), *n* = 8 (group IV).

The grip strength of the forelimbs (peak force) was measured in all animals on the start of the experiment and then once a week using a grip strength meter for rats and mice (model 47200; Ugo Basile, Gemonio VA, Italy). The rat, after grasping the apparatus bar, was pulled by the tail, until the pulling force overcame the grip strength; the peak pull-force achieved by the forelimbs was measured. Data were analyzed and monitored on a computer (DCA software, Ugo Basile, Gemonio VA, Italy).

The rats were fasted overnight after the last administration of phloridzin or the vehicle. The following day, the rats were anesthetized with the i.p. administration of the mixture of ketamine and xylazine, and sacrificed by cardiac exsanguination. After the cessation of vital functions, internal organs (liver, thymus, uterus and kidneys), muscles (musculus tibialis anterior, musculus soleus, musculus gastrocnemius) and bones (right and left tibias, right and left femurs, L-4 vertebra) were isolated. The soft organs were weighed on an Ohaus analytical balance model Adventurer Pro type AV264CM (OHAUS Europe GmbH, Greifensee, Switzerland). After cleaning from soft tissues, the left femurs and tibias, and L-4 vertebras, were also weighed. The long bones were measured (length, diameter in the mid-length) using a digital caliper (VOREL 15240, Toya). The left femurs and tibias, and the proximal part of the right femurs (cut in the middle of the bone length) were protected from drying by wrapping in saline soaked gauze and stored below −20 °C [42]. Blood serum was also frozen until subjected to further measurements.

### 2.2. Biochemical Studies

Serum levels of osteocalcin (a marker of bone formation) and C-terminal telopeptide of type I collagen (CTX-I; a marker of bone resorption) were determined using enzyme immunoassays (Rat-MID Osteocalcin EIA and RatLaps (CTX-I) EIA, respectively, Immunodiagnostic Systems Ltd., Boldon, Tyne and Wear, UK). Insulin concentrations were measured using Mercodia Ultrasensitive Rat Insulin ELISA (Mercodia AB, Uppsala, Sweden). The measurements were performed according to the manufacturers’ instructions, using a microplate reader Stat Fax 2100 (Awareness Technology, Inc., Palm City, FL, USA). Serum concentrations of glucose, fructosamine, triglycerides and uric acid in the rat serum were measured spectrophotometrically, using Biosystems S.A., Costa Brava, Barcelona, Spain, kits. Serum concentrations of HDL and LDL cholesterol were determined spectrophotometrically, using Pointe Scientific, Inc., Canton, MI, USA, kits. The spectrophotometric measurements were performed using a Tecan Infinite M200 PRO plate reader with Magellan 7.2 software (Tecan Austria GmbH, Grödig, Austria), following the manufacturers’ instructions. In order to determine insulin resistance, HOMA-IR (homeostasis model assessment estimate of insulin resistance) was calculated, according to the formula: HOMA-IR = (fasting insulin (mIU/L) × fasting glucose (mg/dL))/405. Moreover, total oxidant status (TOS) was measured according to the method of Erel [43].

### 2.3. Bone Density and Composition Studies

Bone density in the left tibias, left femurs, and L-4 vertebras was evaluated based on Archimedes’ principle [44]. The measurements were made using an analytical balance Adventurer Pro type AV264CM with the density determination kit (OHAUS Europe GmbH, Greifensee, Switzerland). The bones were then lyophilized for 10 days using FreeZone 6 lyophilizer (temperature: −51 °C, pressure: 0.03 mBa; Labconco, Kansas City, MO, USA) and mineralized at 640 °C for 48 h in a muffle furnace L9/11/C6 (Nabertherm, Lilienthal, Germany). The lyophilized and mineralized bones were weighed to determine the mass and content of bone mineral, water and organic substances in bones, as well as bone mineral density (as the ratio of bone mineral mass to bone volume).

### 2.4. Bone Mechanical Properties Studies

An Instron 3342 500N apparatus (Instron, Norwood, MA, USA) was used for measurements of the bone mechanical properties. The data were analyzed using Bluehill 2 software, version 2.14 (Instron, Norwood, MA, USA). Mechanical properties of the proximal metaphysis of the left tibias and mechanical properties of the diaphysis of the left femurs were determined using three-point bending tests. The displacement rate was 0.01 mm/s and the sampling rate was 100 Hz.

As previously described [38,45,46], the proximal tibial epiphysis was removed from the tibia prior to testing of the mechanical properties of the proximal metaphysis. The load was applied perpendicularly to the long axis of the tibia (3 mm from the proximal edge of the bone). The following parameters were assessed for the tibial metaphysis based on the load-displacement curves obtained for each bone: Young’s modulus and the load, displacement, energy and stress for the yield point (0.05% offset), maximum load point and fracture point. To calculate the intrinsic (i.e., independent of bone size and shape) parameters (stress and Young’s modulus), it was assumed that the tibial metaphysis was a circular beam.

In order to determine the strength of the femoral diaphysis [38,42,46], the load was applied perpendicularly to the diaphysis at the bone mid-length. The points supporting the femur were spaced apart by 16 mm. The mechanical test started after initial preload for stable positioning. The same parameters as for tibial metaphysis were assessed, assuming that the femoral diaphysis was an elliptical pipe. The internal and external diameters of the right femoral diaphysis were measured in histological preparations of the mid-length transverse cross-section of the right femur, using OsteoMeasure system (OsteoMetrics Inc., Decatur, GA, USA), consisting of a Carl Zeiss ImagerA1 microscope connected with an Olympus DP71 camera and a computer with OsteoMeasure XP 1.3.0.1 software (OsteoMetrics Inc., Decatur, GA, USA) for histomorphometric measurements and a Wacom graphics tablet model Cintiq 22HD.

Mechanical properties of the femoral neck were studied using a compression test [38,46]. The load was applied to the head of the femur along the long axis of the bone. The load resulting in the fracture of the femoral neck (maximum load) was measured.

### 2.5. Bone Histomorphometric Studies

Histomorphometric measurements of the transverse cross-sections of the tibial and femoral diaphysis were conducted on undecalcified, unstained slides, prepared as previously described [38,47], whereas the measurements of the longitudinal cross-sections of the femoral metaphysis and epiphysis were carried out on decalcified preparations stained with hematoxylin and eosin. All measurements were made using the OsteoMeasure system (OsteoMetrics Inc., Decatur, GA, USA).

The American Society for Bone and Mineral Research (ASBMR) standardized nomenclature was used for presentation of the histomorphometric data [48]. The following parameters of cancellous bone of the distal femoral metaphysis and epiphysis were measured: the bone volume to tissue volume ratio (BV/TV), trabecular thickness (Tb.Th), trabecular separation (Tb.Sp), trabecular number (Tb.N). In the longitudinal preparations from the distal femur, the width of the reserve, proliferative and hypertrophic zones of the epiphyseal cartilage [49] was also measured.

In the cortical bone (tibial and femoral diaphysis), transverse cross-sectional area of the whole diaphysis (Tt.Ar), transverse cross-sectional area of the marrow cavity (Ma.Ar) and the Ma.Ar/Tt.Ar ratio were determined. Moreover, an attempt to measure the periosteal and endosteal transverse growth in the tibial and femoral diaphysis was made. However, tetracycline labels could be observed only in the preparations of the healthy controls, making impossible the direct evaluation of phloridzin effects on those parameters in diabetic rats.

### 2.6. Statistical Analysis

Results are presented as the mean ± standard error of the mean (SEM). Statistical analysis was carried out with the use of one-way analysis of variance (ANOVA) followed by Fisher’s Least Significant Difference (LSD) test (Statistica 13.1; StatSoft Polska Sp. z o.o., Kraków, Poland). The results obtained in phloridzin-treated rats (group III and IV) were compared with those of the HFD/STZ control rats (group II). Results from all groups were also compared with those of the healthy control rats (group I). *p* values < 0.05 were considered significant.

## 3. Results

### 3.1. Effects of Phloridzin on the Body Mass, Non-Fasting Blood Glucose Concentration and Mass of Selected Internal Organs in HFD/STZ Rats

**Figure 1:**
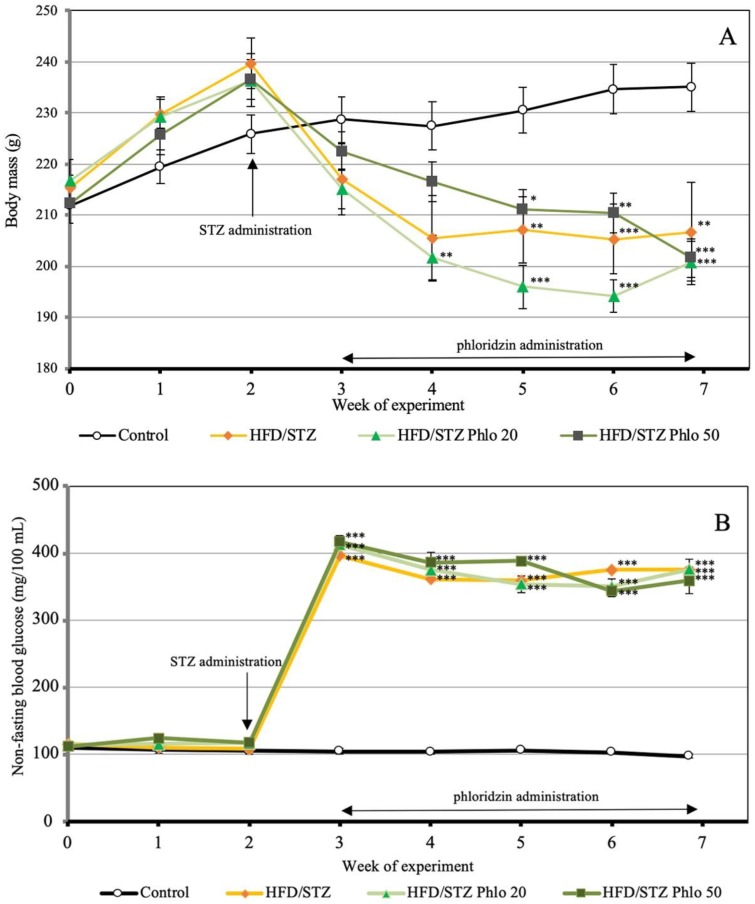
Effect of phloridzin administered orally (20 and 50 mg/kg daily for four weeks) on the body mass (**Panel A**) and non-fasting blood glucose concentration (**Panel B**) in rats with diabetes induced by high-fat diet (HFD) and streptozotocin (STZ). Results are presented as means ± standard error of the mean (SEM) (*n* = 8–11). HFD/STZ—control diabetic rats, HFD/STZ Phlo 20—diabetic rats treated with phloridzin at a dose of 20 mg/kg p.o. daily, HFD/STZ Phlo 50—diabetic rats treated with phloridzin at a dose of 50 mg/kg p.o. daily. The final measurements of the body mass and blood glucose concentration were made before the last phloridzin or vehicle administration. One-way analysis of variance (ANOVA) followed by Fisher’s LSD test was used for evaluation of the significance of the results. * *p* < 0.05, ** *p* < 0.01, *** *p* < 0.001—in comparison with the healthy control rats.

At the start of the experiment (baseline conditions) there were no differences between the rats of all groups concerning the body mass, blood glucose concentration and the grip strength (Figure 1; results for the grip strength not shown). High-fat diet (HFD) induced faster body mass gain in the first two weeks of the experiment in comparison to the rats fed a standard laboratory diet (Figure 1 Panel A). Streptozotocin administration at a dose of 40 mg/kg i.p. two weeks after the introduction of HFD induced diabetes with non-fasting blood glucose concentrations >300 mg/100 mL (Figure 1 Panel B). The body mass of the control diabetic rats after the administration of streptozotocin was lower than that of the healthy control rats through the whole experiment. The mass of the liver and kidneys of HFD/STZ rats significantly increased and the mass of the uterus decreased (insignificantly) in comparison to the healthy controls (Table 1). Administration of phloridzin (20 and 50 mg/kg p.o. daily) for four weeks did not affect the non-fasting blood glucose (Figure 1 Panel B) and body mass in the diabetic rats (Figure 1 Panel A). The uterus mass after administration of phloridzin at a dose of 20 mg/kg p.o. daily further decreased. Phloridzin at a dose of 50 mg/kg p.o. daily strongly tended to decrease the liver mass in the HFD/STZ rats.

### 3.2. Effects of Phloridzin on the Serum Biochemical Parameters in HFD/STZ Rats

Diabetes induced profound disorders of the serum biochemical parameters connected with glucose metabolism (increases in the fasting glucose level and insulin resistance; a strong tendency to increase the fructosamine level) and lipid metabolism (increases in the triglyceride, HDL cholesterol and LDL cholesterol concentrations, and a tendency to decrease the HDL/LDL cholesterol ratio) in relation to the healthy controls (Table 2). The concentration of uric acid also strongly tended to increase. In the control diabetic rats, the serum concentration of a biochemical marker of bone resorption (CTX-I) increased, and that of a marker of bone formation (osteocalcin)—decreased. Administration of phloridzin at a dose of 20 mg/kg p.o. daily to HFD/STZ rats did not significantly affect the above-mentioned serum parameters, in relation to the diabetic controls. Administration of phloridzin at a dose of 50 mg/kg p.o. daily slightly counteracted some effects of diabetes on the serum biochemical parameters in HFD/STZ rats (LDL cholesterol and osteocalcin concentrations were not statistically different from the healthy controls any more, and the HDL/LDL cholesterol ratio increased in relation to the diabetic control rats and returned to the normal values; furthermore, the uric acid concentration normalized). Administration of phloridzin at the lower dose did not affect the total oxidant status (TOS) in diabetic rats, whereas this parameter was decreased (insignificantly) after administration of phloridzin at the higher dose.

### 3.3. Effects of Phloridzin on the Skeletal Muscle Mass and Strength in HFD/STZ Rats

In the HFD/STZ control rats, the mass of the skeletal muscle decreased (m. soleus—insignificantly; Table 3). Also, the grip strength of the HFD/STZ controls tended to decrease in comparison with the healthy controls. Phloridzin at a dose of 20 mg/kg p.o. daily deepened the damaging impact of diabetes on muscle mass and strength; the m. tibialis anterior mass was significantly decreased in comparison to the HFD/STZ controls. All muscle parameters became significantly decreased in relation to the healthy controls. Similar, but slighter effects on the muscle mass and strength were observed after administration of phloridzin at a dose of 50 mg/kg p.o. daily.

### 3.4. Effects of Phloridzin on the Bone Mass, Bone Macrometric Parameters, Density and Composition in HFD/STZ Rats

Diabetes did not affect bone mass and macrometric parameters, however bone density and mineralization (mass of bone mineral/bone mass ratio) in the long bones decreased in relation to the healthy controls (Table 4, data for the tibia not shown). There was no significant effect of diabetes on bone mass, density and mineralization in the L-4 vertebra (not shown). Phloridzin at a dose of 20 mg/kg p.o. daily strongly tended to decrease the length of the femur in the diabetic rats (Table 4). Phloridzin at both doses did not affect bone mass, bone density, bone mineral density and composition of bones (Table 4; data for the tibia and L-4 vertebra not shown) in comparison with the HFD/STZ controls.

### 3.5. Effects of Phloridzin on Bone Histomorphometric Parameters in HFD/STZ Rats

Diabetes induced slight damaging effect on the cancellous bone microstructure in HFD/STZ rats. The BV/TV value strongly tended to decrease and Tb.Th significantly decreased in the femoral metaphysis in relation to the healthy controls (Table 5). Administration of phloridzin (20 and 50 mg/kg p.o. daily) did not affect the histomorphometric parameters of the femoral metaphysis. There was no effect of the diabetes and phloridzin on the histomorphometric parameters of the femoral epiphysis (not shown).

In the distal epiphyseal cartilage of the femur of HFD/STZ rats, the width of the hypertrophic zone decreased in comparison with the control rats. Phloridzin at both doses intensified the effect of diabetes on the width of the hypertrophic zone of the epiphyseal cartilage.

In compact bone of the femoral diaphysis, diabetes induced an increase in Tt.Ar (Table 5). Also, Ma.Ar and the Ma.Ar/Tt.Ar ratio strongly tended to increase. In the tibia, diabetes tended to increase Tt.Ar and Ma.Ar (not shown). Administration of phloridzin at a dose of 20 mg/kg p.o. daily to HFD/STZ rats counteracted the effect of diabetes on Tt.Ar in the femur and tibia. The femoral Tt.Ar of those rats was significantly decreased, and the tibial Tt.Ar tended to decrease in comparison with the HFD/STZ controls. Phloridzin at a dose of 50 mg/kg p.o. daily did not significantly affect the histomorphometric parameters of cortical bone in the femur, whereas it strongly tended to decrease the Tt.Ar in the tibia, in comparison with the HFD/STZ controls.

### 3.6. Effects of Phloridzin on Bone Mechanical Properties in HFD/STZ Rats

Mechanical properties of the tibial metaphysis (mostly cancellous bone) were strongly worsened in HFD/STZ rats. Yield point load, maximum load and fracture load, as well as the corresponding values of energy and stress, were significantly reduced, whereas a decrease in Young’s modulus was statistically insignificant in the HFD/STZ control rats in relation to the healthy controls (Table 6; the data for energy, displacement and stress not shown). Diabetes did not significantly affect mechanical properties of the femoral diaphysis (compact bone), and of the femoral neck built of compact and cancellous bone (Table 7). Administration of phloridzin did not significantly affect mechanical properties of cancellous and compact bone of the tibia and femur, respectively, in diabetic rats. Although some bone parameters tended to be changed, no consistent tendencies were demonstrated.

## 4. Discussion

Various antidiabetic drugs exert differential effects on the skeletal system. For example, metformin and incretin-based therapies may favorably affect the skeletal system, whereas thiazolidinediones are known to exert deleterious effects on bones [50]. SGLT2 inhibitors are suspected to unfavorably affect the skeletal system, although the results of clinical observations are inconsistent [50,51,52]. Phloridzin, an apple polyphenol and a precursor of SGLT2 inhibitors, was reported to exert hypoglycemic effects [6,9,14,17,18,19,53]. In the present study, the effects of administration of phloridzin at two doses on the musculoskeletal system of rats with experimentally induced type 2 diabetes were investigated. Both doses were above those achievable in normal human diet, taking into account the mentioned in the Introduction phloridzin content in apple fruits [6] and the fact that the rat metabolism is faster than in humans [54].

In the present study, the HFD/STZ rats developed hyperglycemia, with increased insulin resistance (HOMA-IR) and a tendency to increase the fructosamine concentration in comparison with the healthy controls. The severity of hyperglycemia was very similar to those reported by others (for review, see Reference [36]). As opposed to humans with type 2 diabetes, the body mass decreased in the HFD/STZ diabetic rats. The liver mass increased, indicating disorders of its function, and increases in the serum triglyceride and cholesterol concentrations were observed. An increase in the kidney mass was accompanied by a tendency to increase the uric acid concentration, suggesting impaired kidney function [55]. Administration of phloridzin at a dose of 20 mg/kg p.o. did not favorably affect the above-mentioned changes induced by diabetes. Although phloridzin at a dose of 50 mg/kg p.o. also did not affect the biochemical parameters of carbohydrate metabolism, some parameters of lipid metabolism were improved, namely the HDL/LDL cholesterol ratio was normalized, and the LDL cholesterol concentration decreased in relation to the diabetic controls. Favorable effects of phloridzin on the lipid profile were already demonstrated in experimental conditions in rats (5–40 mg/kg p.o. for three weeks) [17] and mice (20 mg/kg p.o. for 10 weeks [18] or 0.02% in the diet for 16 weeks [19]). The liver mass also tended to decrease. The fact that we did not observe the decreasing effect of phloridzin on the glucose level might have resulted from the study design. For example, in some studies that demonstrated this effect, the measurements were performed shortly after phloridzin administration in rats (5–40 mg/kg p.o.) [17] and mice (about 50 mg/kg p.o.) [14], as opposite to our measurements performed 24 h after the last administration, or phloridzin was administered with the diet in mice (0.02%, 0.1%, 0.5%) [9,19]. It seems important that no effect of phloridzin on fructosamine level was noted in the present study. Since fructosamine is a measure of non-enzymatic glycation of plasma proteins and reflects changes of blood glucose concentration over time [56], the results indicate low potential of phloridzin as an antidiabetic agent.

The development of the metabolic changes induced by T2D was accompanied by severe disorders within the musculoskeletal system. Most of the musculoskeletal measurements performed in the study were direct ones (bone and muscle mass, bone macrometric parameters, density, mechanical properties and histomorphometric parameters), providing reliable results. The HFD/STZ diabetes induced profound changes in the serum bone turnover markers, indicating a strong increase in bone resorption and decrease in bone formation. Although the experimental diabetes did not significantly affect bone mass and macrometric parameters, bone density and content of mineral substances in the femur significantly decreased. Disorders in cancellous bone histomorphometric parameters (decreases in Tb.Th and BV/TV in the femoral metaphysis) led to significant worsening of the mechanical properties of the proximal tibial metaphysis, consistently with the previous results obtained in diabetic rats [57] and the increased fracture risk in humans [22,23]. Diabetes affected also the epiphyseal cartilage, decreasing the width of hypertrophic zone. In compact bone, no significant effects of diabetes on the mechanical properties were demonstrated. However, the diabetic rats had increased transverse cross-sectional area of the femoral diaphysis, which corresponds with the increased bone mass sometimes observed in humans with type 2 diabetes [22]. The HFD/STZ diabetes unfavorably affected also the skeletal muscle mass and strength (as assessed in the grip test), consistently with numerous previous observations in experimental models of diabetes and in patients [23,25].

The effects of phloridzin administered at doses of 20 and 50 mg/kg p.o. on the musculoskeletal system were differential.

Phloridzin at a dose of 20 mg/kg p.o. definitely unfavorably affected the musculoskeletal system of the diabetic rats. Both muscle mass and strength decreased in relation to the diabetic controls. In the skeletal system, phloridzin at this dose induced a decrease in compact bone formation (as assessed by measurements of the transverse cross-sectional area of the femoral diaphysis). However, it must be stated that this effect counteracted the effect of diabetes. Moreover, phloridzin at the lower dose inhibited the longitudinal bone growth, since the length of the femur decreased, and the diabetes-induced decrease in the width of the hypertrophic zone of the epiphyseal cartilage was intensified in comparison with the diabetic controls. It may be speculated that decreases in muscle mass and bone formation and growth induced by phloridzin in the lower dose may result from its effects via PPARγ receptors, since phloretin, an aglycone of phloridzin, was reported to increase their expression and transcriptional activity [58,59]. Thiazolidinediones, agonists of those receptors, exert unfavorable effect on the musculoskeletal system, leading to the increased fracture risk [23,50] and skeletal muscle injury [60]. What is more, the HFD/STZ rats receiving phloridzin at a dose of 20 mg/kg p.o. had decreased the uterus mass; such an effect was not observed in rats administered phloridzin at the higher dose. Phloretin was reported to have some affinity for estrogen receptors and exert favorable effects in the skeletal system of estrogen-deficient mice [29,31]. It is possible that phloretin may act in a way similar to selective estrogen receptor modulators (SERMs), as an antagonist in the uterus and possibly in the muscle, and an agonist in bone.

Phloridzin at the higher dose (50 mg/kg p.o.) did not affect most of the investigated muscle and bone parameters in the diabetic rats, with the exception of normalization of the serum level of osteocalcin (a marker of bone formation). Osteocalcin and insulin are both engaged in regulation of energy metabolism; osteocalcin favors insulin secretion and increases insulin tissue sensitivity [61,62]. The increase in osteocalcin level may contribute to favorable effects of phloridzin at a dose of 50 mg/kg p.o. on the serum biochemical parameters of lipid metabolism, disordered in HFD/STZ diabetic rats.

In the previous studies, phloridzin was reported to exert some favorable effect on the skeletal system of rats and mice [27,28]. Besides different experimental models, the differences between the results of those studies and the present study may be an effect of species differences [28] and the doses used [27]. In the only previous study on the influence of phloridzin on the rat skeletal system [27], the dose of phloridzin was much higher (0.25% in the diet, i.e., about 200 mg/kg body mass) than those used in the present study. The lack of a dose-dependent phloridzin effect in this study strongly supports the hypothesis that the mechanisms of phloridzin action at smaller and larger doses are different. Phloridzin at a dose of 20 mg/kg p.o. exerted more severe unfavorable effects on the skeletal muscle mass and strength, and decreased the longitudinal and transverse growth of the femur in the diabetic rats. It may be speculated that, at higher doses, the antioxidant effect of phloridzin was revealed, which inhibited the adverse effects on muscle mass and bone histomorphometric and macrometric parameters, observed at the lower dose. This notion is supported by the results of measurements of serum total oxidant status, which demonstrated its lowering (albeit insignificant) by phloridzin at a dose of 50 mg/kg p.o.

Summing up, our results demonstrated some favorable effects of high-dose phloridzin on the serum biochemical parameters of lipid metabolism. The effects, however, were not demonstrated for the lower dose. Moreover, the lower dose unfavorably affected musculoskeletal system, which should arise concern about the safety of the use of apple-based supplements in diabetes.

The main limitation of the study was lack of randomization of the animals at the start of the experiment, since it might have affected the effect size [63]. Another limitation of the study was that it was performed in one experimental model only; other phloridzin musculoskeletal effects cannot be excluded. The main advantage of the study is that the very wide range of methods used in the experiments that allowed to demonstrate consistent damaging effect of phloridzin at a dose of 20 mg/kg p.o. daily on the musculoskeletal system in rats. However, since species differences may occur, the results of the study cannot be directly generalized to humans. The mechanisms responsible for the observed phloridzin effects need to be elucidated in further studies. The study indicates also the need for future clinical or epidemiological observations.

## 5. Conclusions

The study demonstrated differential effects of phloridzin in a model of T2D in rats, depending on the dose. The results indicate that phloridzin should not be used in the treatment and prophylaxis of osteoporosis in diabetes. The long-term intake of products containing high doses of phloridzin may adversely affect the musculoskeletal system.

## Figures and Tables

**Table 1:** Effect of phloridzin administered orally (20 and 50 mg/kg daily for four weeks) on the mass of selected internal organs in rats with diabetes induced by high-fat diet (HFD) and streptozotocin (STZ).

Parameter/Group		Control	HFD/STZ	HFD/STZ Phlo 20	HFD/STZ Phlo 50
Mass	Uterus (g)	0.417 ± 0.046	0.299 ± 0.064	0.184 ± 0.034 **	0.309 ± 0.048
	Liver (g)	5.494 ± 0.128	7.004 ± 0.164 ***	6.919 ± 0.169 ***	6.596 ± 0.088 ***
	Kidneys (g)	1.445 ± 0.038	1.789 ± 0.055 ***	1.766 ± 0.062 ***	1.729 ± 0.064 ***

Results are presented as means ± standard error of the mean (SEM) (*n* = 8–11). HFD/STZ—control diabetic rats, HFD/STZ Phlo 20—diabetic rats treated with phloridzin at a dose of 20 mg/kg p.o. daily, HFD/STZ Phlo 50—diabetic rats treated with phloridzin at a dose of 50 mg/kg p.o. daily. One-way analysis of variance (ANOVA) followed by Fisher’s LSD test was used for evaluation of the significance of the results. ** *p* < 0.01, *** *p* < 0.001—in comparison with the healthy control rats.

**Table 2:** Effect of phloridzin administered orally (20 and 50 mg/kg daily for four weeks) on the serum biochemical parameters in rats with diabetes induced by high-fat diet (HFD) and streptozotocin (STZ).

Parameter/Group	Control	HFD/STZ	HFD/STZ Phlo 20	HFD/STZ Phlo 50
Glucose (mg/100 mL)	74.85 ± 8.88	290.36 ± 15.52 ***	311.30 ± 21.95 ***	279.27 ± 10.95 ***
Insulin (μg/L)	0.115 ± 0.019	0.118 ± 0.020	0.105 ± 0.030	0.106 ± 0.014
HOMA-IR	0.464 ± 0.095	2.076 ± 0.306 ***	2.124 ± 0.673 ***	1.835 ± 0.239 ***
Fructosamine (mmol/L)	1.86 ± 0.06	2.21 ± 0.14	2.15 ± 0.12	2.04 ± 0.08
Triglycerides (mg/100 mL)	11.34 ± 1.44	41.79 ± 13.95	53.44 ± 20.24	22.56 ± 3.10
HDL cholesterol (mg/100 mL)	57.7 ± 2.3	119.3 ± 10.8 ***	124.2 ± 10.1 ***	108.2 ± 7.5 ***
LDL cholesterol (mg/100 mL)	5.05 ± 0.60	27.93 ± 10.30 *	27.22 ± 8.30 *	10.23 ± 2.75
HDL/LDL cholesterol ratio	13.44 ± 1.77	8.34 ± 2.09	7.77 ± 1.42 *	13.54 ± 2.00
Uric acid (μmol/L)	29.57 ± 1.91	54.74 ± 13.53	51.29 ± 11.41	33.10 ± 1.85
TOS (μmol H_2_O_2_ Equiv./L)	7.67 ± 1.05	10.93 ± 3.31	11.00 ± 2.43	5.93 ± 0.87
CTX-I (ng/mL)	18.21 ± 1.10	39.97 ± 7.78 **	52.06 ± 5.60 ***	34.68 ± 4.20 *
Osteocalcin (ng/mL)	175.5 ± 12.1	116.6 ± 12.5 **	97.7 ± 10.7 ***	162.4 ± 26.6

Results are presented as means ± standard error of the mean (SEM) (*n* = 8–11). HFD/STZ—control diabetic rats, HFD/STZ Phlo 20—diabetic rats treated with phloridzin at a dose of 20 mg/kg p.o. daily, HFD/STZ Phlo 50—diabetic rats treated with phloridzin at a dose of 50 mg/kg p.o. daily. HOMA-IR—homeostasis model assessment estimate of insulin resistance. HDL—high density lipoprotein. LDL—low density lipoprotein. TOS—total oxidant status. CTX-I—C-terminal type I collagen fragments. One-way analysis of variance (ANOVA) followed by Fisher’s LSD test was used for evaluation of the significance of the results. * *p* < 0.05, ** *p* < 0.01, *** *p* < 0.001—in comparison with the healthy control rats.

**Table 3:** Effect of phloridzin administered orally (20 and 50 mg/kg daily for four weeks) on the skeletal muscle mass and strength in rats with diabetes induced by high-fat diet (HFD) and streptozotocin (STZ).

Parameter/Group	Control	HFD/STZ	HFD/STZ Phlo 20	HFD/STZ Phlo 50
Musculus gastrocnemius mass (g)	1.063 ± 0.042	0.925 ± 0.055 *	0.795 ± 0.048 ***	0.875 ± 0.046 **
Musculus soleus mass (g)	0.090 ± 0.006	0.079 ± 0.004	0.074 ± 0.001 **	0.078 ± 0.002 *
Musculus tibialis anterior mass (g)	0.425 ± 0.011	0.373 ± 0.019 **	0.318 ± 0.006 *** ##	0.377 ± 0.011 **
Grip strength (N)	4.50 ± 0.28	3.73 ± 0.33	3.15 ± 0.34 **	3.20 ± 0.31 **

Results are presented as means ± standard error of the mean (SEM) (*n* = 8–11). HFD/STZ—control diabetic rats, HFD/STZ Phlo 20—diabetic rats treated with phloridzin at a dose of 20 mg/kg p.o. daily, HFD/STZ Phlo 50—diabetic rats treated with phloridzin at a dose of 50 mg/kg p.o. daily. The grip strength was measured before the last phloridzin/vehicle administration. One-way analysis of variance (ANOVA) followed by Fisher’s LSD test was used for evaluation of the significance of the results. * *p* < 0.05, ** *p* < 0.01, *** *p* < 0.001—in comparison with the healthy control rats, ## *p* < 0.01—in comparison with the HFD/STZ control rats.

**Table 4:** Effect of phloridzin administered orally (20 and 50 mg/kg daily for four weeks) on macrometric parameters, mass, density and mineralization of the femur in rats with diabetes induced by high-fat diet (HFD) and streptozotocin (STZ).

Parameter/Group	Control	HFD/STZ	HFD/STZ Phlo 20	HFD/STZ Phlo 50
Bone length (mm)	33.82 ± 0.32	33.70 ± 0.15	33.07 ± 0.22	33.61 ± 0.20
Bone mean diameter (mm)	3.41 ± 0.02	3.47 ± 0.04	3.41 ± 0.03	3.45 ± 0.03
Bone mass (g)	0.680 ± 0.013	0.690 ± 0.009	0.656 ± 0.014	0.684 ± 0.012
Bone density (g/cm^3^)	1.586 ± 0.007	1.546 ± 0.011 **	1.549 ± 0.011 **	1.535 ± 0.008 ***
Mass of bone mineral (g)	0.330 ± 0.007	0.321 ± 0.005	0.311 ± 0.007	0.317 ± 0.007
Bone mineral density (g/cm^3^)	0.722 ± 0.009	0.676 ± 0.017 *	0.680 ± 0.015 *	0.663 ± 0.013 **
Mass of bone mineral/bone mass ratio	0.486 ± 0.003	0.466 ± 0.007 *	0.474 ± 0.007	0.464 ± 0.006 *
Mass of bone water/bone mass ratio	0.276 ± 0.003	0.289 ± 0.007	0.280 ± 0.008	0.290 ± 0.008
Mass of bone organic substances/bone mass ratio	0.238 ± 0.002	0.246 ± 0.005	0.245 ± 0.004	0.247 ± 0.003

Results are presented as means ± standard error of the mean (SEM) (*n* = 8–11). HFD/STZ—control diabetic rats, HFD/STZ Phlo 20—diabetic rats treated with phloridzin at a dose of 20 mg/kg p.o. daily, HFD/STZ Phlo 50—diabetic rats treated with phloridzin at a dose of 50 mg/kg p.o. daily. One-way analysis of variance (ANOVA) followed by Fisher’s LSD test was used for evaluation of the significance of the results. * *p* < 0.05, ** *p* < 0.01, *** *p* < 0.001—in comparison with the healthy control rats.

**Table 5:** Effect of phloridzin administered orally (20 and 50 mg/kg daily for four weeks) on histomorphometric parameters of the distal femoral metaphysis, epiphyseal cartilage and diaphysis in rats with diabetes induced by high-fat diet (HFD) and streptozotocin (STZ).

Parameter/Group		Control	HFD/STZ	HFD/STZ Phlo 20	HFD/STZ Phlo 50
Metaphysis	BV/TV (%)	44.84 ± 2.11	37.01 ± 2.13	39.58 ± 2.88	37.89 ± 2.54
	Tb.Th (µm)	64.56 ± 2.58	52.47 ± 3.12 *	54.14 ± 4.04 *	52.00 ± 3.44 *
	Tb.Sp (µm)	80.30 ± 4.31	89.67 ± 4.45	83.77 ± 6.35	86.23 ± 5.87
	Tb.N (1/mm)	6.95 ± 0.18	7.08 ± 0.20	7.33 ± 0.27	7.32 ± 0.30
Epiphyseal cartilage	Reserve zone width (µm)	27.05 ± 1.21	27.88 ± 3.77	21.67 ± 1.04	24.15 ± 1.37
	Proliferative zone width (µm)	29.55 ± 0.94	26.54 ± 2.83	21.29 ± 0.76 ***	23.34 ± 1.36 **
	Hypertrophic zone width (µm)	32.52 ± 1.71	25.64 ± 1.84 **	21.69 ± 0.54 ***	23.45 ± 0.85 ***
Diaphysis	Tt.Ar (mm^2^)	8.318 ± 0.116	8.711 ± 0.107 *	8.141 ± 0.147 *##*	8.365 ± 0.166
	Ma.Ar (mm^2^)	3.069 ± 0.071	3.471 ± 0.099	3.192 ± 0.129	3.219 ± 0.118
	Ma.Ar/Tt.Ar	0.369 ± 0.007	0.398 ± 0.009	0.391 ± 0.010	0.384 ± 0.009

Results are presented as means ± standard error of the mean (SEM) (*n* = 8–11). HFD/STZ—control diabetic rats, HFD/STZ Phlo 20—diabetic rats treated with phloridzin at a dose of 20 mg/kg p.o. daily, HFD/STZ Phlo 50—diabetic rats treated with phloridzin at a dose of 50 mg/kg p.o. daily. BV/TV—bone volume/tissue volume ratio; Tb.Th—trabecular thickness; Tb.Sp—trabecular separation; Tb.N—trabecular number; Tt.Ar—transverse cross-sectional area of the whole diaphysis; Ma.Ar—transverse cross-sectional area of the marrow cavity; Ma.Ar/Tt.Ar—transverse cross-section of the marrow cavity/diaphysis area ratio. One-way analysis of variance (ANOVA) followed by Fisher’s LSD test was used for evaluation of the significance of the results. * *p* < 0.05, ** *p* < 0.01, *** *p* < 0.001—in comparison with the healthy control rats. ## *p* < 0.01—in comparison with the HFD/STZ control rats.

**Table 6:** Effect of phloridzin administered orally (20 and 50 mg/kg daily for four weeks) on mechanical properties of the proximal tibial metaphysis in rats with diabetes induced by high-fat diet (HFD) and streptozotocin (STZ).

Parameter/Group	Control	HFD/STZ	HFD/STZ Phlo 20	HFD/STZ Phlo 50
Young’s modulus (MPa)	3425 ± 195	3045 ± 188	2754 ± 309	2483 ± 309
Yield point load (N)	73.5 ± 11.1	31.6 ± 2.0 ***	44.2 ± 4.9 **	34.7 ± 4.0 ***
Maximum load (N)	113.2 ± 7.3	70.8 ± 4.5 ***	74.0 ± 4.1 ***	75.0 ± 3.8 ***
Fracture load (N)	86.0 ± 7.4	53.3 ± 5.9 ***	56.3 ± 3.8 ***	59.4 ± 5.6 **

Results are presented as means ± standard error of the mean (SEM) (*n* = 8–11). HFD/STZ—control diabetic rats, HFD/STZ Phlo 20—diabetic rats treated with phloridzin at a dose of 20 mg/kg p.o. daily, HFD/STZ Phlo 50—diabetic rats treated with phloridzin at a dose of 50 mg/kg p.o. daily. One-way analysis of variance (ANOVA) followed by Fisher’s LSD test was used for evaluation of the significance of the results. ** *p* < 0.01, *** *p* < 0.001—in comparison with the healthy control rats.

**Table 7:** Effect of phloridzin administered orally (20 and 50 mg/kg daily for four weeks) on mechanical properties of the femur in rats with diabetes induced by high-fat diet (HFD) and streptozotocin (STZ).

Parameter/Group		Control	HFD/STZ	HFD/STZ Phlo 20	HFD/STZ Phlo 50
Diaphysis	Young’s modulus (MPa)	8443 ± 311	7896 ± 277	9272 ± 436	8121 ± 501
	Yield point load (N)	76.9 ± 6.3	80.7 ± 3.5	82.4 ± 4.1	80.2 ± 1.9
	Maximum load (N)	127.9 ± 4.3	135.3 ± 4.2	125.7 ± 3.7	130.4 ± 4.5
	Fracture load (N)	126.7 ± 4.2	135.1 ± 4.3	121.7 ± 3.4	130.4 ± 4.5
Neck	Maximum load (N)	86.1 ± 3.6	95.3 ± 5.6	86.5 ± 2.9	89.9 ± 3.1

Results are presented as means ± standard error of the mean (SEM) (*n* = 8–11). HFD/STZ—control diabetic rats, HFD/STZ Phlo 20—diabetic rats treated with phloridzin at a dose of 20 mg/kg p.o. daily, HFD/STZ Phlo 50—diabetic rats treated with phloridzin at a dose of 50 mg/kg p.o. daily. One-way analysis of variance (ANOVA) followed by Fisher’s LSD test was used for evaluation of the significance of the results.
